# MicroRNA-17-5p-activated Wnt/β-catenin pathway contributes to the progression of liver fibrosis

**DOI:** 10.18632/oncotarget.6447

**Published:** 2015-12-02

**Authors:** Fujun Yu, Zhongqiu Lu, Kate Huang, Xiaodong Wang, Ziqiang Xu, Bicheng Chen, Peihong Dong, Jianjian Zheng

**Affiliations:** ^1^ Department of Infectious Diseases, The First Affiliated Hospital of Wenzhou Medical University, Wenzhou, China; ^2^ Emergency Department, The First Affiliated Hospital of Wenzhou Medical University, Wenzhou, China; ^3^ Department of Pathology, The First Affiliated Hospital of Wenzhou Medical University, Wenzhou, China; ^4^ Institute of Organ Transplantation, The First Affiliated Hospital of Wenzhou Medical University, Wenzhou, China; ^5^ Key Laboratory of Surgery, The First Affiliated Hospital of Wenzhou Medical University, Wenzhou, China

**Keywords:** microRNA-17-5p, Wnt/β-catenin pathway, hepatic stellate cells, Salvianolic acid B, Wnt inhibitory factor 1 (WIF1), Pathology Section

## Abstract

Aberrant Wnt/β-catenin pathway contributes to the development of liver fibrosis. MicroRNAs (MiRNAs) are found to act as regulators of the activation of hepatic stellate cell (HSC) in liver fibrosis. However, whether miRNAs activate Wnt/β-catenin pathway in activated HSCs during liver fibrosis is largely unknown. In this study, we found that Salvianolic acid B (Sal B) treatment significantly inhibited liver fibrosis in CCl_4_-treated rats, HSC-T6 cells and rat primary HSCs, resulting in the suppression of type I collagen and alpha-smooth muscle actin. Also, Sal B suppressed HSC activation and cell proliferation *in vitro*. Interestingly, Sal B treatment induced the inactivation of Wnt/β-catenin pathway, with an increase in P-β-catenin and Wnt inhibitory factor 1 (WIF1). We demonstrated that the anti-fibrotic effects caused by Sal B were, at least in part, via WIF1. Moreover, our study revealed that miR-17-5p was reduced *in vivo* and *in vitro* after Sal B treatment. As confirmed by luciferase activity assays, WIF1 was a direct target of miR-17-5p. Notably, the suppression of HSCs induced by Sal B was almost inhibited by miR-17-5p mimics. Collectively, we demonstrated that miR-17-5p activates Wnt/β-catenin pathway to result in HSC activation through inhibiting WIF1 expression.

## INTRODUCTION

Liver fibrosis, a major cause of morbidity and mortality worldwide, represents the common responses of the liver to infectious, toxic or metabolic agents and is a big medical problem [1]. Liver fibrosis is characterized by an excessive deposition of extracellular matrix (ECM) proteins in liver, mainly synthesized by activated hepatic stellate cells (HSCs). The excess deposition of ECM disrupts the normal architecture of the liver resulting in pathophysiological damage to the organ, which eventually undergoes the liver fibrosis-cirrhosis [2]. During liver injury, quiescent HSCs are exposed to inflammatory and profibrogenic factors, and transdifferentiate into myofibroblast-like cells that are characterized by expression of alpha-smooth muscle actin (α-SMA), contributing to the progression of liver fibrosis [3]. Therefore, HSCs, the major mesenchymal cells in liver, are widely accepted as playing a critically important role in liver fibrosis [4]. However, the underlying molecular mechanisms responsible for the proliferation and activation of HSCs are still not completely understood.

Aberrant Wnt/β-catenin signaling has been shown to be involved in the development of organ fibrosis including liver fibrosis [5, 6]. Sustained Wnt/β-catenin pathway activation contributes to HSC activation and mediates HSC proliferation, resolution, and ECM accumulation [5, 7, 8]. It has been demonstrated that Wnt antagonists such as secreted Frizzled-related proteins (sFRPs), Wnt inhibitory factor 1 (WIF1) and secreted Dickkopf (DKK) family (DKK1-4) can attenuate hepatic fibrosis *via* inhibiting Wnt/β-catenin pathway, suggesting Wnt/β-catenin pathway may be a novel therapeutic target in liver fibrosis [9, 10].

MicroRNAs (miRNAs) are short 20-22 nucleotides and act as negative regulators of gene expression by inhibiting protein translation or inducing mRNA degradation [11]. Growing evidence has demonstrated that miRNAs are involved in the control of a wide range of biological functions and processes such as development, differentiation, metabolism, carcinogenesis, and immune response [12]. HSCs could be activated or suppressed by miRNAs, suggesting that miRNAs act as HSC regulators in liver fibrosis. For instance, over-expression of miR-146a suppresses transforming growth factor-beta (TGF-β)-induced HSC proliferation, and increases HSC apoptosis *via* its target Smad4 [4]. Recently, miR-17-5p, a member of the miR-17-92 cluster, is often up-regulated in many malignancies including hepatocellular carcinoma (HCC) and functions as an oncogenic miRNA [13, 14]. MiR-17-5p is not only involved in cell functions such as proliferation and migration but also a key regulator of the G1/S phase cell cycle transition [15]. Our previous study demonstrated that over-expression of miR-17-5p promotes HSC proliferation and activation [16].

In China, traditional Chinese medicine is often used to treat hepatic fibrosis because of patients' trust in traditional Chinese medicine. For example, Fuzheng Huayu recipe (FZHY), a formula with anti-hepatic fibrosis activity, is often used as an anti-liver fibrotic product in China [17, 18]. It has been demonstrated that Radix Salviae miltiorrhizae is the main effective herb in FZHY. Salvianolic acid B (Sal B), one of the water soluble components from Radix Salviae miltiorrhizae, has good action against hepatic fibrosis in animal model and patients with chronic hepatitis B [19, 20]. But so far, the mechanisms for the anti-fibrotic effects of Sal B have not been well elucidated.

## RESULTS

### Sal B inhibited liver fibrosis *in vivo* and *in vitro*

**Figure 1:**
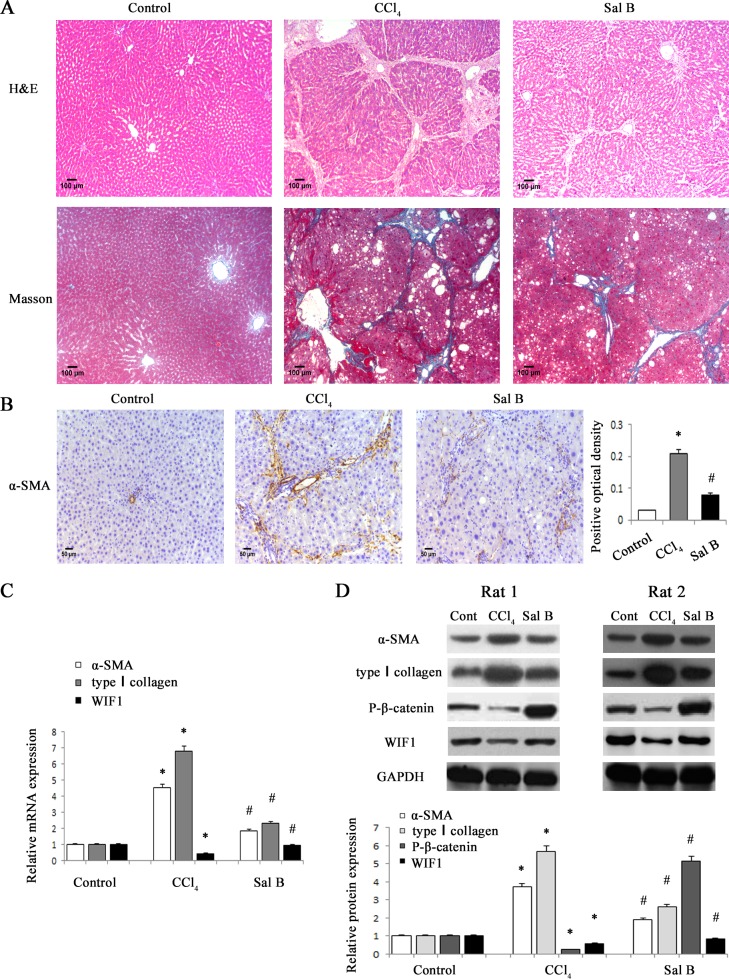
Sal B significantly ameliorated CCl_**4**_-induced liver fibrosis in rats **A.** H&E staining (×100) and Masson staining (×100) for assessing liver fibrosis. **B.** The levels of α-SMA were analyzed by immunohistochemistry in CCl_4_-treated rats after Sal B treatment. Representative views from each group are presented (original magnification, ×10). **C.** The mRNA levels of α-SMA, type I collagen and WIF1 were detected in CCl_4_-treated rats after Sal B treatment. **D.**The protein levels of α-SMA, type I collagen, P-β-catenin and WIF1 were detected in CCl_4_-treated rats after Sal B treatment. GAPDH was used as internal control. Each value is the mean ± SD of three experiments. *P < 0.05 compared with the control and ^#^*P* < 0.05 compared with the CCl_4_ group.

**Figure 2:**
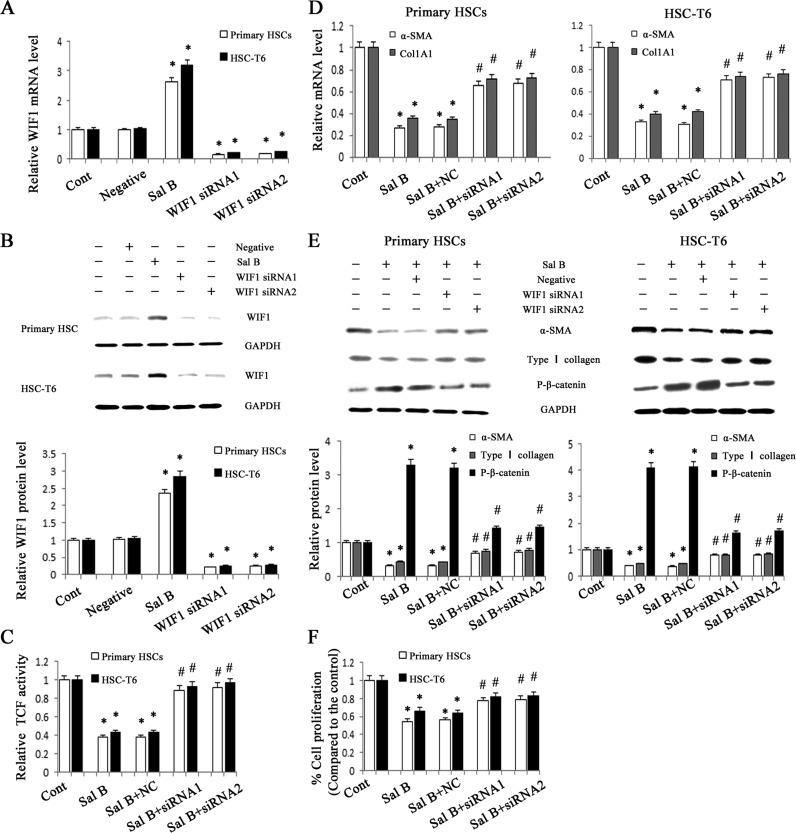
Roles of WIF1 in the anti-fibrotic effects induced by Sal B HSC-T6 cells and primary HSCs were transfected with WIF1 siRNA for 48 h and/or treated with Sal B for 48 h. **A.** The mRNA expression of WIF1 was analyzed by real-time PCR. **B.** The protein expression of WIF1 was analyzed by western blotting. GAPDH was used as internal control. **C.** Reduced TCF activity by Sal B was restored by WIF1 siRNA. **D.** The decreased mRNA levels of α-SMA and Col1A1 induced by Sal B were blocked down by WIF1 siRNA. **E.** The effects of Sal B on the protein levels of α-SMA, type I collagen and P-β-catenin were attenuated by WIF1 siRNA. GAPDH was used as internal control. **F.** The reduction of cell proliferation induced by Sal B was inhibited by WIF1 siRNA. Each value is the mean ± SD of three experiments. *P < 0.05 compared with the control and ^#^*P* < 0.05 compared with the Sal B group.

It has been demonstrated that Sal B can inhibit liver fibrosis [20, 21]. To confirm the effects of Sal B on rat liver fibrosis induced by carbon tetrachloride (CCl_4_), the degree of rat liver fibrosis was determined by hematoxylin and eosin (H&E) staining and Masson staining. As indicated by H&E and Masson staining, CCl_4_ caused prominent hepatic steatosis, necrosis, and formation of regenerative nodules in rat liver tissues, which was ameliorated by Sal B (Figure 1A). Immunohistochemical results demonstrated that the increased α-SMA levels in CCl_4_-treated rats were reduced by Sal B (*P* < 0.05, Figure 1B). Next, the effects of Sal B on the mRNA and protein levels of α-SMA and type I collagen were analyzed in rat liver tissues by real-time PCR and western blotting, respectively. Our results showed that the increased levels of α-SMA and type I collagen caused by CCl_4_ were inhibited by Sal B (Figure 1C and Figure 1D). To further investigate the anti-fibrotic effects of Sal B *in vitro*, the levels of α-SMA and type I collagen were analyzed in HSC-T6 cells and primary HSCs after Sal B treatment. Compared with the control, the levels of α-SMA and type I collagen were reduced by Sal B in HSC-T6 cells (Figure 2D and Figure 2E). Similar results were also observed in primary HSCs. As shown in the MTT assay, the growth rate was reduced to 53.8% and 65.6%, respectively, in Sal B-treated primary HSCs and HSC-T6 cells compared to untreated cells (Figure 2F). These data confirmed that Sal B could inhibit liver fibrosis.

### Sal B induced the inactivation of Wnt/β-catenin pathway and the up-regulation of WIF1 expression

Aberrant Wnt/β-catenin pathway is involved in the development of organ fibrosis [22, 23]. To explore whether Wnt/β-catenin pathway is involved in the effects of Sal B on liver fibrosis, the protein levels of P-β-catenin were detected *in vivo* and *in vitro* after Sal B treatment. The results showed that Sal B caused a significant increase in the phosphorylation of β-catenin in CCl_4_-treated rats, HSC-T6 cells and primary HSCs (Figure 1D and Figure 2E). Sal B treatment also resulted in a reduced TCF activity in HSC-T6 and primary HSCs (Figure 2C). To further study whether the suppression of Wnt signaling caused by Sal B is associated with the increased expressions of Wnt signaling inhibitors, the mRNA expressions of Wnt signaling inhibitors including WIF1, sFRP1, sFRP2 and DKK1-4, were detected in CCl_4_-treated rats, HSC-T6 cells and primary HSCs after Sal B treatment. It was found that the mRNA level of WIF1 was increased by Sal B whereas others not (Figure 1C, Figure 2A and Supporting Information Figure S1). Immunoblot analysis further confirmed that Sal B caused an increase in WIF1 protein *in vivo* and *in vitro* (Figure 1D and Figure 2B). Therefore, WIF1 was chosen for the next experiments. These results indicated that Sal B caused the suppression of Wnt/β-catenin pathway with an increase in the levels of P-β-catenin and WIF1.

### WIF1 up-regulation caused by Sal B suppressed HSC activation

Previous studies have reported that WIF1 is a potent Wnt pathway antagonist [9]. To explore whether WIF1 is involved in the anti-fibrotic effect induced by Sal B, WIF1 siRNA was transfected into HSC-T6 cells and primary HSCs to silent WIF1. The blockade of WIF1 with WIF1 siRNA successfully inhibited the mRNA and protein levels of WIF1 in HSC-T6 cells and primary HSCs (Figure 2A and Figure 2B). Next, the TCF activity and the levels of P-β-catenin in Sal B-treated cells with WIF1 siRNA were examined. TCF reporter activity assay showed that the TCF activity decreased by Sal B was reversed by WIF1 siRNA in HSC-T6 cells and primary HSCs (Figure 2C). Moreover, the enhanced P-β-catenin levels caused by Sal B were reversed by WIF1 siRNA (Figure 2E). Of note, the effects of Sal B on the mRNA and protein levels of α-SMA and type I collagen in HSCs were blocked down by WIF1 siRNA (Figure 2D and Figure 2E). The reduction of cell proliferation caused by Sal B treatment was also blocked down by the silencing of WIF1 expression (Figure 2F). These findings demonstrated that WIF1 was involved in the anti-fibrotic effect induced by Sal B.

### MiR-17-5p was involved in Sal B effects and promoted the activation of HSCs

**Figure 3:**
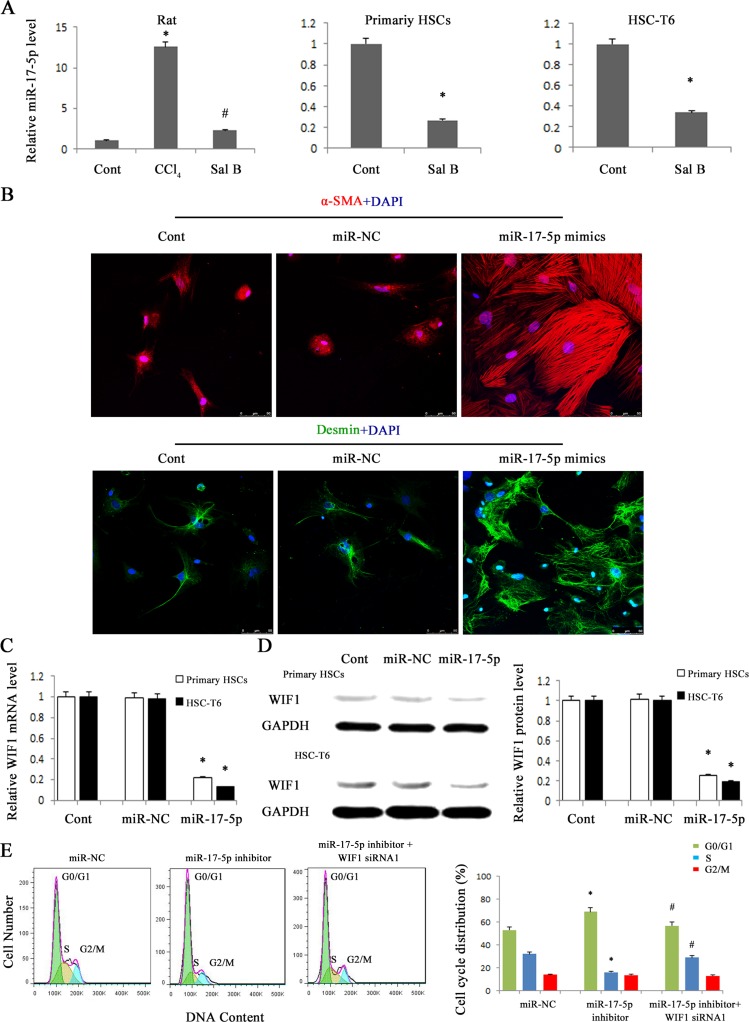
miR-17-5p over-expression promoted the activation of HSCs and contributed to the reduction of WIF1 levels HSC-T6 cells and primary HSCs were transfected with miR-17-5p mimics or miR-NC for 48 h. **A.** The down-regulation of miR-17-5p expression was found in CCl_4_-treated rats, HSC-T6 cells and primary HSCs after Sal B treatment. **B.** Immunofluorescence staining for α-SMA (red) and desmin (green) were evaluated by confocal laser microscopy. DAPI stained nuclei blue. Scale bar, 50 μm. **C.** The mRNA expression of WIF1 was analyzed by real-time PCR in HSCs treated with miR-17-5p mimics. **D.** The protein expression of WIF1 was analyzed by western blotting in HSCs treated with miR-17-5p mimics. GAPDH was used as internal control. **E.** Effect of WIF1 siRNA on the cell cycle in HSCs transfected with miR-17-5p inhibitor. The primary HSCs were transfected with miR-17-5p inhibitor for 48 h and then transfected with WIF1 siRNA for an additional 48 h. Each value is the mean ± SD of three experiments. *P < 0.05 compared with the control or miR-NC. ^#^*P* < 0.05 compared with miR-17-5p inhibitor group.

**Figure 4:**
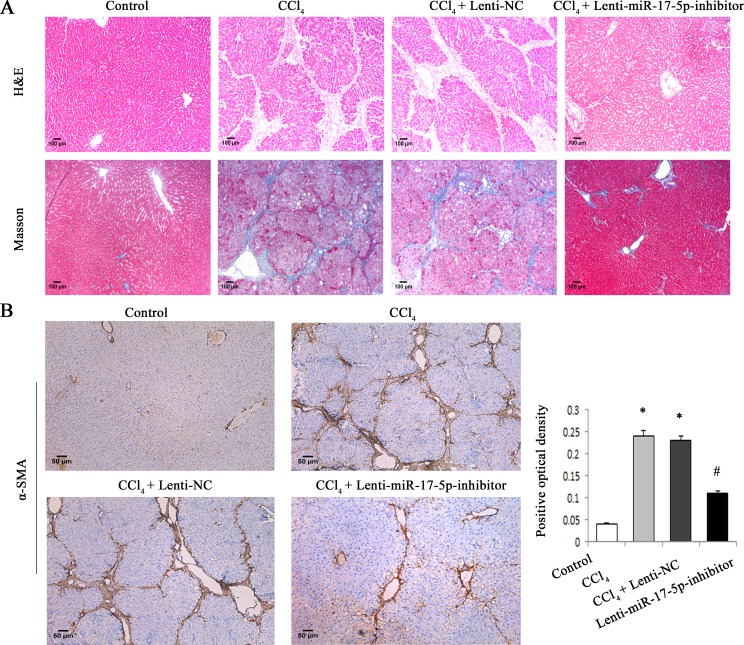
MiR-17-5p inhibitor treatment significantly suppressed rat liver fibrosis caused by CCl_**4**_ **A.** H&E staining (×100) and Masson staining (×100) for assessing liver fibrosis. **B.** The levels of α-SMA were analyzed by immunohistochemistry in CCl_4_-treated rats after miR-17-5p inhibitor treatment. Representative views from each group are presented (original magnification, ×10). Each value is the mean ± SD of three experiments. *P < 0.05 compared with the control and ^#^*P* < 0.05 compared with the CCl_4_ group.

**Figure 5:**
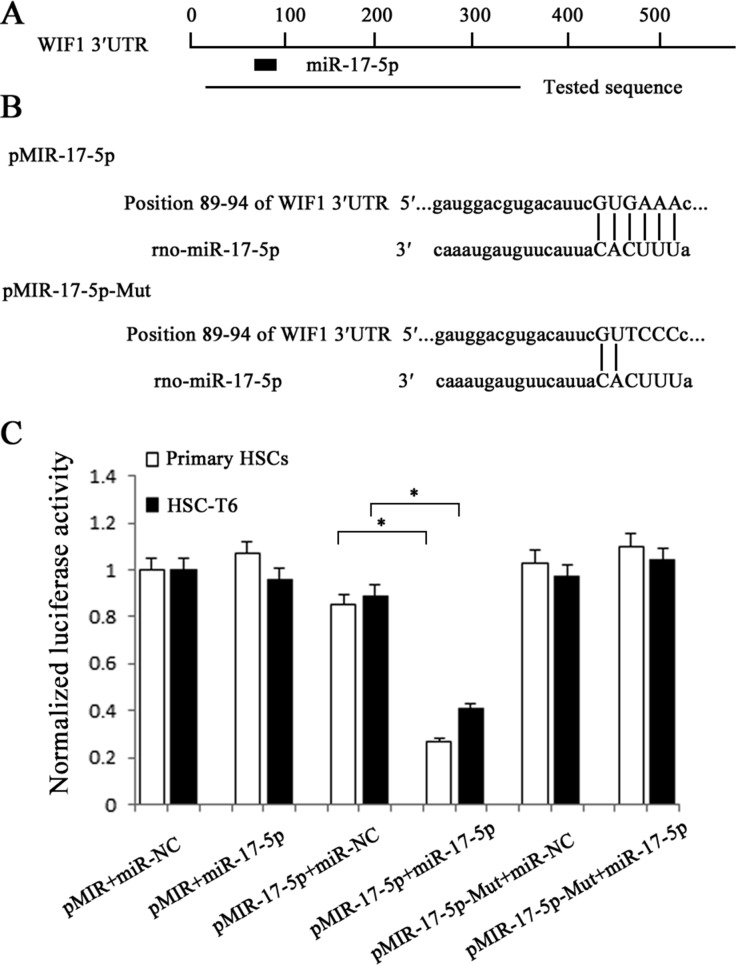
Interaction of miR-17-5p with the 3′UTR of WIF1 **A.** Schematic indication of the miRNA binding sites in the 3′UTR of WIF1 mRNA based on microRNA.org. Black box indicates miR-17-5p and a tested sequence indicates the region that was inserted into the luciferase reporter vector. **B.** Predicted consequential pairing of the target region and miR-17-5p. According to the pairing sites, the corresponding luciferase reporter vectors were named pMIR-17-5p and pMIR-17-5p-Mut. **C.** The HSCs were transfected with pMIR (empty vector), pMIR containing miR-17-5p targeting sequence (pMIR-17-5p) and pMIR with miR-17-5p mutated target sequence (pMIR-17-5p-Mut). The graph shows luciferase activity in cells transfected with pMIR-17-5p or pMIR-17-5p-Mut. It also shows cotransfection of miR-17-5p precursor or miR-NC. *P < 0.05.

To determine whether WIF1 expression is inhibited by miRNAs, we searched for predictable miRNAs that could bind with 3′untranslated region (3′UTR) of rat WIF1 mRNA using microRNA.org (http://www.microrna.org/microrna/home.do). As a result, miR-16, miR-17-5p, miR-20a, miR-20b-5p, miR-21 and miR-199-5p were extracted as candidates. The results showed that only miR-17-5p was reduced *in vivo* and *in vitro* after Sal B treatment (Figure 3A and Supporting Information Figure S1). Therefore, miR-17-5p was selected for further experiments. To investigate the effects of miR-17-5p over-expression on the activation of HSCs, immunofluorescence staining for α-SMA and the HSC marker desmin was examined in primary HSCs. We found that the levels of desmin (green) and α-SMA (red) were increased by miR-17-5p over-expression (Figure 3B). These data indicated that miR-17-5p promoted the activation of HSCs. Given that WIF1 was predicted as a putative target of miR-17-5p (Figure 5A and Figure 5B), we hypothesized that miR-17-5p might promote hepatic fibrosis *via* inhibiting WIF1 expression. Next, the mRNA and protein levels of WIF1 were examined in cells transfected with miR-17-5p mimics. The results showed that both the mRNA and protein levels of WIF1 were reduced by miR-17-5p mimics (Figure 3C and Figure 3D). We also examined the effects of miR-17-5p inhibitor and the silencing of WIF1 on cell cycle. Cell cycle analysis revealed that compared with the control, miR-17-5p inhibitor suppressed a proportion of cells in the S phase and increased the number of cells in the G0/G1 phase, suggesting that miR-17-5p inhibitor contributed to the inactivation of HSCs (Figure 3E). However, the effects of miR-17-5p inhibitor on cell cycle were blocked down by WIF1 siRNA (Figure 3E). Notably, as shown in H&E and Masson staining, miR-17-5p inhibitor treatment significantly suppressed liver fibrosis caused by CCl_4_ (Figure 4A). Immunohistochemical results additionally confirmed that the levels of α-SMA increased in CCl_4_-treated rats were attenuated by miR-17-5p inhibitor treatment (*P* < 0.05, Figure 4B). All these results suggested that miR-17-5p could promote the activation of HSCs *via* the down-regulation of WIF1.

### WIF1 is a target of miR-17-5p

To further investigate whether WIF1 is the direct downstream target of miR-17-5p, the sequence of 3′UTR of WIF1 mRNA target region was cloned into pMIR-REPORT^TM^ Luciferase plasmid (Figure 5A and Figure 5B). The construct was cotransfected into HSCs along with miR-17-5p precursor or miRNA negative control (miR-NC). β-gal reporter control plasmid was cotransfected to monitor transfection efficiency. Our results showed that miR-17-5p precursor significantly reduced luciferase activity driven by the wild-type 3′UTR of WIF1 compared to miR-NC in HSCs. Meanwhile, the luciferase activities of mutated type WIF1 3′UTR and empty vector were not inhibited by miR-17-5p precursor (Figure 5C). These results confirmed that WIF1 was a direct target of miR-17-5p.

### MiR-17-5p activated HSCs *via* Wnt/β-catenin pathway

**Figure 6:**
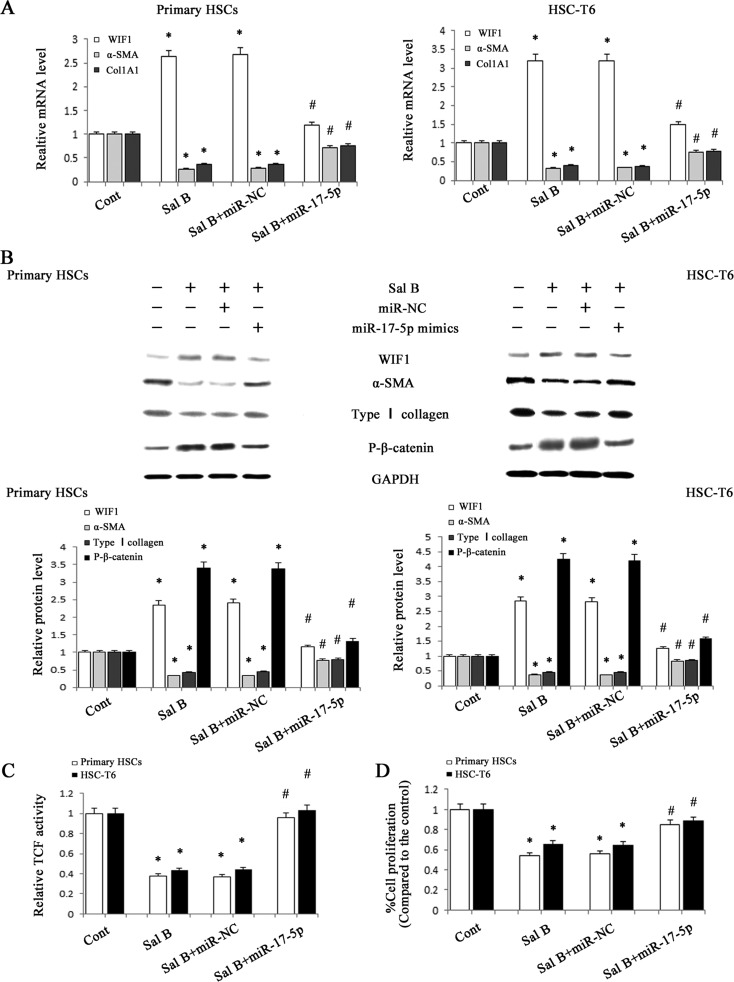
Roles of miR-17-5p in the anti-fibrotic effects induced by Sal B HSC-T6 cells and primary HSCs were transfected with miR-17-5p mimics for 48 h and treated with Sal B for additional 48 h. **A.** The mRNA expressions of WIF1, α-SMA and Col1A1 were analyzed by real-time PCR. **B.** The protein expressions of WIF1, α-SMA, type I collagen and P-β-catenin were analyzed by western blotting. GAPDH was used as internal control. **C.** The reduction of TCF activity induced by Sal B was restored by miR-17-5p mimics. **D.** The reduction of cell proliferation induced by Sal B was restored by miR-17-5p mimics. Each value is the mean ± SD of three experiments. *P < 0.05 compared with the control and ^#^*P* < 0.05 compared with the Sal B group.

Our previous study showed that WIF1 was a direct target of miR-17-5p and the levels of miR-17-5p were reduced by Sal B in HSCs. To further investigate whether miR-17-5p is involved in the anti-fibrotic effects of Sal B, we transfected miR-17-5p mimics into Sal B-treated cells. The effects of Sal B on the levels of P-β-catenin, α-SMA and type I collagen were attenuated by miR-17-5p mimics (Figure 6A and Figure 6B). Notably, increased WIF1 levels in Sal B-treated cells were additionally reversed by miR-17-5p mimics. Moreover, the reduction of TCF activity and cell proliferation rate induced by Sal B were almost blocked down by miR-17-5p mimics (Figure 6C and Figure 6D). All these results indicated that the reduction of miR-17-5p level in the treatment of Sal B contributed to the suppression of activated HSCs and miR-17-5p promoted the activation of Wnt/β-catenin pathway *via* inhibiting WIF1 level.

## DISCUSSION

Activation of HSCs to myofibroblast-like cells is the pivotal event in the initiation and progression of hepatic fibrosis [24]. HSC activation is characterized by enhanced cell proliferation, over-production of ECM, and de novo synthesis of α-SMA [25]. In this study, we found that Wnt/β-catenin pathway was attenuated by Sal B *via* restoration of WIF1 and inhibition of miR-17-5p, with a reduction in TCF activity and an increase in P-β-catenin level. Owing to the suppression of Wnt/β-catenin pathway, HSC activation was inhibited, leading to the reduction of HSC proliferation, ECM proteins and α-SMA expression. The silencing of WIF1 blocked down the anti-fibrotic effects of Sal B and WIF1 was a direct target of miR-17-5p. These data revealed that Sal B suppresses HSC activation, at least in part, through inhibiting miR-17-5p-activated Wnt/β-catenin pathway.

It has been reported that HSC activation can be suppressed by Sal B treatment. Wang *et al*. showed that Sal B prevents the progression of liver angiogenesis and alleviates liver fibrosis *via* NF-κB signaling [26]. Li *et al*. reported that Sal B may exert an anti-hepatic fibrosis effect *via* down-regulating angiotensin II signaling in HSC activation [20]. Another study demonstrated that Sal B prevents HSC activation through TGF-β signaling pathway, i.e. inhibiting TGF-β1 expression, activity of TβR-I kinase and Smads phosphorylation [27]. In our study, it was found that Sal B could inhibit HSC activation, at least in part, *via* suppressing Wnt/β-catenin pathway. To our knowledge, this is the first report to show that miR-17-5p-activated Wnt/β-catenin pathway is involved in the effects of Sal B.

**Figure 7:**
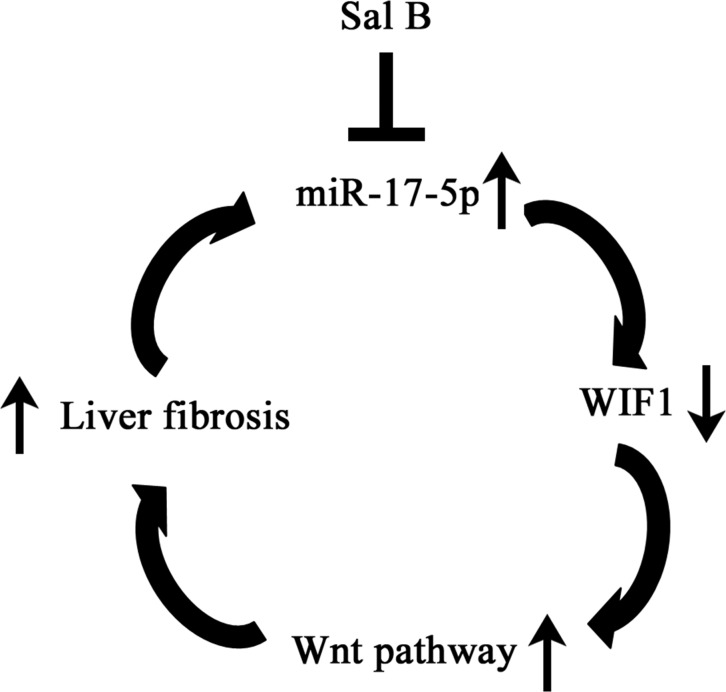
The signaling pathway was discovered in activated HSCs after Sal B treatment Sal B induces miR-17-5p down-regulation, WIF1 up-regulation, the inactivation of Wnt pathway, resulting in the inhibition of the activated HSCs.

MiR-17-5p was described as an oncogenic miRNA in cancers. For example, Li *et al*. found that miR-17-5p is able to enhance cell proliferation by promoting G1/S transition of the cell cycle and suppressing apoptosis in ovarian cancer cell lines [28]. Moreover, emerging studies show that miR-17-5p over-expression promotes HCC development and the inhibition of miR-17-5p results in the suppression of HCC proliferation and migration [29, 30]. Notably, elevated serum miR-17-5p has also been reported to correlate with the poor prognosis in HCC patients [31]. In this study, miR-17-5p over-expression increased the levels of α-SMA and desmin in primary HSCs. Furthermore, miR-17-5p was involved in the regulation of cell cycle and inhibited the effects of Sal B on cell proliferation. Notably, the inhibition of miR-17-5p suppressed CCl_4_-induced liver fibrosis. All the data suggest that miR-17-5p over-expression contributes to the activation of HSCs, which is also consistent with our previous study [16]. We previously showed that miR-17-5p promotes HSC proliferation and activation, at least in part, *via* reduction of Smad7 [16]. Herein, we demonstrated that WIF1 is a new target of miR-17-5p. In addition, it was found that all the anti-fibrotic effects of Sal B could be blocked down by miR-17-5p mimics. Taken together, our data suggested that the activation of HSCs was suppressed by Sal B *via* miR-17-5p and WIF1 (Figure 7). However, the mechanism of the direct regulation of miR-17-5p by Sal B still remains unclear and further studies are warranted.

In conclusion, we demonstrate that Sal B down-regulates miR-17-5p expression, leading to the restoration of WIF1 and the inhibition of Wnt/β-catenin signaling, which contributes to the suppression of activated HSCs. Our results not only provide a new insight of the role of miRNA-activated Wnt/β-catenin signaling in liver fibrosis, but also show a new anti-fibrotic mechanism of Sal B.

## MATERIALS AND METHODS

### Materials

CCl_4_ was obtained from Sigma (St Louis, MO, USA). Antibodies against type I collagen, WIF1 and P-β-catenin were obtained from Abcam (Cambridge, MA, USA). Antibodies against α-SMA and GAPDH were purchased from Santa Cruz Biotechnology (Santa Cruz, CA, USA).

### Cell culture

Rat HSC-T6 cell line was obtained from Research of the Chinese Academy of Medical Sciences (Beijing, China). Cells were grown in DMEM containing 10% fetal bovine serum (FBS) (Gibco, Carlsbad, CA) and maintained in a 37°C incubator with 5% CO_2_. HSC-T6 cells and primary rat HSCs were both treated with 10 μmol/L Sal B for 48 h, which is a safe dose [27].

### Isolation and culture of rat HSCs

Adult male Sprague-Dawley rats (body weight, 400-500 g) were used for HSC isolation as described previously [32]. The primary HSCs were studied at day 3 after isolation throughout all the studies. The purity of cultures was confirmed by immunocytochemical staining for α-SMA and the purity reached > 98%.

### CCl_4_ liver injury model

Adult male Sprague-Dawley (180-220 g) rats were provided by the Experimental Animal Center of Wenzhou Medical University. Liver fibrosis was induced by administration of 2 ml of CCl_4_/olive oil (1:1, v/v)/kg body weight by intraperitoneal injection twice weekly for up to 6 weeks [33]. Thirty rats were randomly divided into three groups, including olive oil, CCl_4_ plus oral phosphate-buffered saline (PBS) and CCl_4_ plus oral Sal B (10 mg /kg), respectively. The experimental protocol was approved by the Institutional Animal Committee of Wenzhou Medical College. All animals received care in accordance with ‘Guide for the Care and Use of Laboratory Animals’. Rats were sacrificed under anesthesia at the end of six weeks and the livers were removed for further analysis. The liver tissues were used for H&E staining and Masson staining by fixation with 10 % formalin.

### Lentivirus production and transfection

The lentiviral vector containing negative control (Lenti-NC) and lentiviral miR-17-5p inhibitor (Lenti-miR-17-5p-inhibitor) were obtained from Shanghai GeneChem. Rats were treated with olive oil (*n* = 6), CCl_4_ (*n* = 6), CCl_4_ plus Lenti-NC (*n* = 6) and CCl_4_ plus Lenti-miR-17-5p-inhibitor (*n* = 6). Lenti-miR-17-5p-inhibitor or Lenti-NC was injected *via* the tail vein only once at three weeks after CCl_4_ injection (1×10^9^ transducing unit/rat). After the following 3-weeks CCl_4_ treatment, the rats were sacrificed.

### RNA interference analysis

RNA interference experiments were performed before the treatment of Sal B using Lipofectamine 2000 (Invitrogen Carlsbad, CA, USA) in accordance with the manufacturer's instructions. siRNA oligonucleotides against WIF1 or scrambled sequences (Table. S1) were synthesized by Gene Pharma (Shanghai, China) and transfected in HSC-T6 cell and primary HSCs for 48 h.

### miRNA transfection

Cells were seeded in a 6-well plate at a density of 1×10^6^ cells per well. Then, medium was replaced with Opti-MEM (Invitrogen, USA) and cells were transfected with miR-17-5p mimics (60 nM) and miR-NC (60 nM) (GenePharma, China) using Lipofectamine 2000 for 48 h. After 6 h of transfection, the medium was replaced with DMEM containing 10 % FBS.

### Immunofluorescence microscopy

Cells were seeded on 18-mm cover glasses and fixed in an acetic acid: ethanol (1:3) solution for 5 min at −20°C. Nonspecific binding was blocked with 5% goat and horse serum/PBS for 1 h at room temperature. Then, cells were incubated with primary antibodies against α-SMA or desmin (Abcam), followed by fluorescein-labeled secondary antibody (1:50 dilution; Dianova) [34]. The nuclei were stained with 4,6-diamidino-2-phenylindole (DAPI). The slides were washed twice with PBS, covered with DABCO (Sigma-Aldrich), and examined with confocal laser scanning microscopy (Olympus, Tokyo, Japan) at 488 and 568 nm.

### Immunohistochemistry

Immunohistochemical staining was performed on the sections (3 μm thick) from the liver tissues, as described previously [35, 36]. Briefly, after deparaffinization, hydration, and antigen retrieval, samples were incubated overnight at 4°C with a primary antibody against α-SMA (1:100) and then with a biotinylated secondary antibody. α-SMA expression was visualized by 3,3′-diaminobenzidine tetrahydrochloride (DAB) staining. Slides were counterstained with hematoxylin before dehydration and mounting α-SMA-positive areas within the fibrotic region were then observed. Quantitative analysis was calculated from five fields for each liver slice.

### Quantitative real-time PCR

Total RNA was extracted from tissues and cells using miRNeasy Mini kit (Qiagen, Valencia, CA, USA) according to manufacturer's instructions. Gene expression (Table. S1) was measured by real-time PCR using SYBR Green real-time PCR Master Mix (Toyobo, Osaka, Japan). Moreover, the primers of alpha-1 (I) collagen (Col1A1), α-SMA, GAPDH and U6 were designed as described previously [37]. Expression of mature miRNAs was detected using TaqMan MicroRNA Assay (Applied Biosystems, Foster City, CA). The GAPDH and U6 snRNA levels were used to normalize the relative abundance of mRNAs and miRNAs [38], respectively.

### Western blot analysis

Tissues and cells were lysed with ice-cold lysis buffer (50 mM Tris-HCl, pH 7.4, 100 mM 2-Mercaptoethanol, 2% w/v SDS, 10% glycerol). Total proteins were quantified and separated by SDS-PAGE. Then western blot assay was performed as described previously [39]. The levels of protein were normalized to total GAPDH.

### Cell proliferation assay

Cells were seeded in a 96-well plate at a density of 1×10^3^ cells per well, then cells were transfected with miR-17-5p mimics and miR-NC as described above. Cell proliferation was determined by MTT assay according to the instructions of a MTT cell proliferation assay kit (Beyotime Biotechnology, Jiangsu, China). The optical density was measured at 570 nm on a microplate reader (Bio-Rad 550, USA).

### TCF reporter activity assay

Cells were transiently transfected with TOPFLASH and FOPFLASH (Upstate Biotechnology Inc., Lake Placid, NY, USA) using Lipofectamine 2000. Twenty-four hours after transfection, the cells were harvested and luciferase and Renilla luminescence were measured using the Dual-Luciferase Reporter Assay System (Promega, Wisconsin, WI, USA) on a luminometer (BioTek Instruments, Winooski, VT, USA). TCF reporter activity was presented as the ratio of firefly-to-Renilla luciferase activity.

### Cell cycle analysis

For cell cycle analysis, we performed Cell Cycle Analysis Kit (Beyotime, China). Cells were fixed in 70 % ethanol in PBS at −20°C for 24 h and then labeled with 0.5 ml propidium iodide (PI) staining buffer containing 200 mg/ml RNase A and 50 μg/ml PI at 37°C for 40 min in the dark. Analyses were performed on a BD LSR flow cytometer (BD Biosciences) and experiments repeated at least three times.

### Luciferase activity assay

The 3′UTR region of WIF1 gene was cloned into the pMIR-REPORT™ Luciferase plasmid (Applied Biosystems) to generate pMIR-17-5p and pMIR-17-5p-Mut vectors. WIF1 3′UTR for miR-17-5p forward, 5′-TCGAGTTACGCCGAGTTCAC-3′ and reverse, 5′-GTTTCGCCTCTCTAGGGCTC-3′. Transfection was performed with Lipofectamine 2000 according to the manufacturer's recommendations [40]. pMIR-REPORT β-gal control plasmid was used for transfection normalization. Luciferase values were measured using Dual-Light System (Applied Biosystems).

### Statistical analysis

Data from at least three independent experiments were expressed as the mean ± SD. Statistical analysis was performed using Student's *t*-test and *P* < 0.05 was considered significant. All statistical analyses were performed with SPSS software (version 13; SPSS, Chicago, IL).

## SUPPLEMENTARY MATERIAL FIGURE AND TABLE


