# Supplementary material for: MicroRNA-17-5p-activated Wnt/β-catenin pathway contributes to the progression of liver fibrosis
Source: Oncotarget. 2015 Dec 2;7(1):81–93. doi: 10.18632/oncotarget.6447 (PMC4807984; doi:10.18632/oncotarget.6447)
Supplement: Supplementary file 1 [file oncotarget-07-0081-s001.pdf]

MicroRNA-17-5p-activated Wnt/β-catenin pathway contributes to the progression of liver fibrosis

Supplementary Material

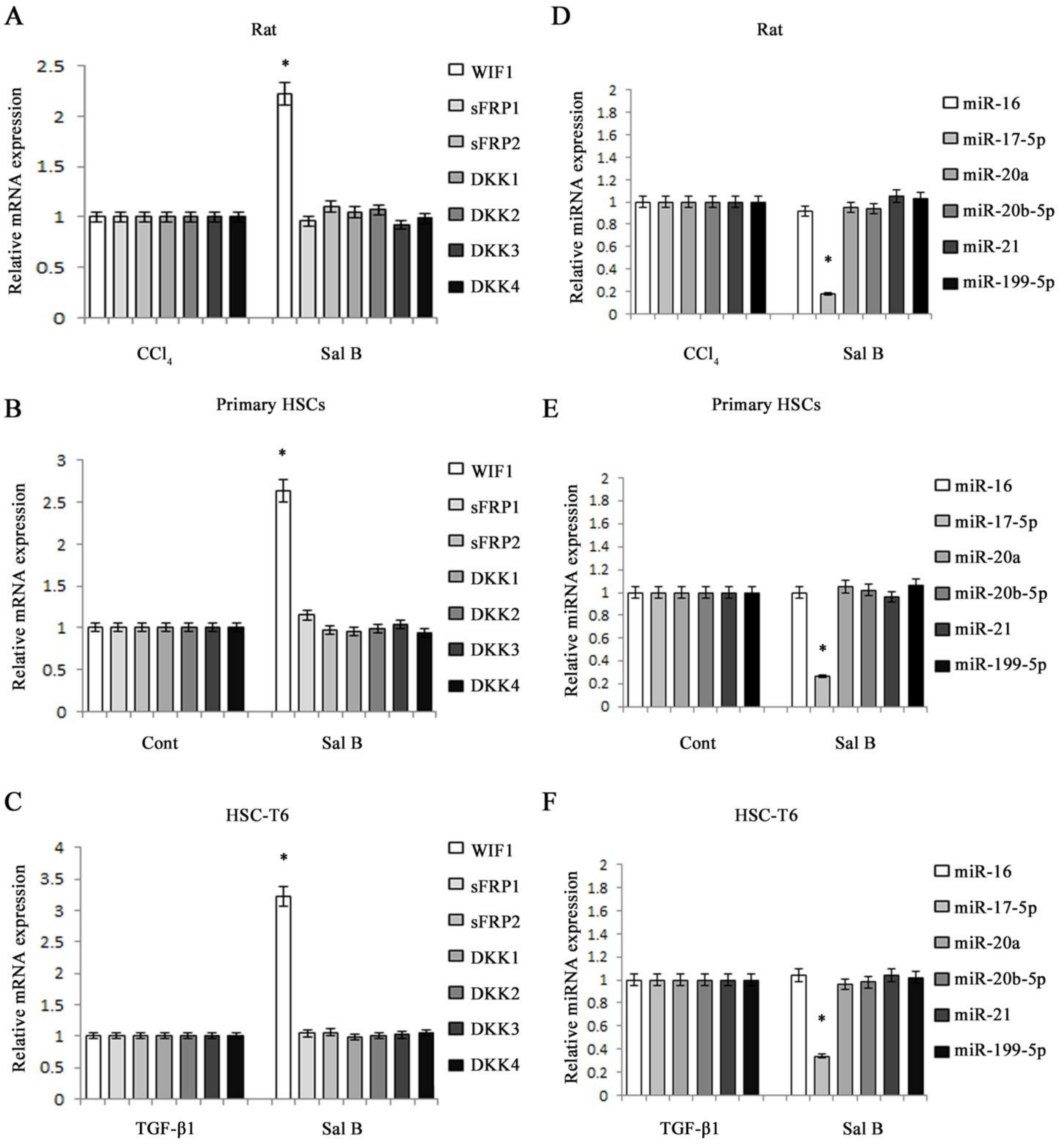

Fig. S1: Expressions of Wnt signaling inhibitors and predictable miRNAs that could bind with 3'UTR of rat WIF1 mRNA using microRNA.org were detected in vivo and in vitro after Sal B treatment. HSC-T6 cells and primary HSCs were treated with Sal B for 48 h. The mRNA expressions of Wnt signaling inhibitors including WIF1, sFRP1, sFRP2 and DKK1-4 were detected in CCl4-treated rats (A), primary HSCs (B) and HSC-T6 cells (C) after Sal B treatment. The expressions of predictable miRNAs including miR-16, miR-17-5p, miR-20a, miR-20b-5p, miR-21 and miR-199-5p were detected in CCl4-treated rats (D), primary HSCs (E) and HSC-T6 cells (F) after Sal B treatment. Each value is the mean ± SD of three experiments. *P <0.05 compared with the control.

Table.S1 Primer sequences

Gene	Forward sequence	Reverse sequence
WIF1	5'-ATAAAAGGTACGGAGCCAGCC-3'	5'-CTGTGAACTCGGCGTAACTC-3'
sFRP1	5'-CCTGGCTCTGTTCCTACAGC-3'	5'-CTGACTCTCCTTGTGCCTGC-3'
sFRP2	5'-GATCACCTCCGTGAAACGGT-3'	5'-CGGAAATGAGGTCGCAGAGT-3'
DKK1	5'-GGTCGTGCTTTCAACGATGG-3'	5'-GCAGGTTCTTGATCGCGTTG-3'
DKK2	5'-AGTACATTTGCCCTCGCACA-3'	5'-AGCCTGGACAGATGACAAGC-3'
DKK3	5'-ATTGGCATGGGGACAAGTAGG-3'	5'-GCTGCCCTGCTGACCTAAAT-3'
DKK4	5'-ACGGTCTGCGTGAATGATGT-3'	5'-TGAGGTCTGTTTTCCTCCGC-3'
WIF1 siRNA1	5'-AUCUUCUUCAAAUCCUAUGAG-3'	5'-CAUAGGAUUUGAAGAAGAUAU-3'
WIF1 siRNA2	5'-ACAUUCACUUCAAAUGCUGCC-3'	5'-CAGCAUUUGAAGUGAAUGUGA-3'
negative control	5'-UUCUCCGAACGUGUCACGUTT-3'	5'-ACGUG ACACGUU CGGAGAATT-3'
